# Alkyladenine DNA glycosylase deficiency uncouples alkylation-induced strand break generation from PARP-1 activation and glycolysis inhibition

**DOI:** 10.1038/s41598-020-59072-6

**Published:** 2020-02-10

**Authors:** Fahad A. Alhumaydhi, Debora de O. Lopes, Diana L. Bordin, Abdullah S. M. Aljohani, Cameron B. Lloyd, Michael D. McNicholas, Larissa Milano, Clara F. Charlier, Izabel Villela, João Antonio P. Henriques, Kathryn E. Plant, Ruan M. Elliott, Lisiane B. Meira

**Affiliations:** 1 Clinical and Experimental Medicine, Faculty of Health and Medical Sciences, University of Surrey, Guildford, UK; 2 Nutritional Sciences, Faculty of Health and Medical Sciences, University of Surrey, Guildford, UK; 3 Centro de Biotecnologia, Universidade Federal do Rio Grande do Sul, Porto Alegre, RS Brazil; 4 Present Address: Department of Medical Laboratories, College of Applied Medical Science, Qassim University, Qassim, Kingdom of Saudi Arabia; 5 Present Address: Department of Clinical Molecular Biology, Akershus University Hospital, Sykehusveien 25, Nordbyhagen, Norway; 6 Present Address: Department of Veterinary Medicine, College of Agriculture and Veterinary Medicine, Qassim University, Qassim, Kingdom of Saudi Arabia; 7 Present Address: Genome Stability Laboratory, CHU de Québec Research Center, HDQ Pavilion, Oncology Division, 9 McMahon, Québec City, QC Canada

**Keywords:** Biochemistry, DNA

## Abstract

DNA alkylation damage is repaired by base excision repair (BER) initiated by alkyladenine DNA glycosylase (AAG). Despite its role in DNA repair, AAG-initiated BER promotes cytotoxicity in a process dependent on poly (ADP-ribose) polymerase-1 (PARP-1); a NAD^+^-consuming enzyme activated by strand break intermediates of the AAG-initiated repair process. Importantly, PARP-1 activation has been previously linked to impaired glycolysis and mitochondrial dysfunction. However, whether alkylation affects cellular metabolism in the absence of AAG-mediated BER initiation is unclear. To address this question, we temporally profiled repair and metabolism in wild-type and *Aag*^−/−^ cells treated with the alkylating agent methyl methanesulfonate (MMS). We show that, although *Aag*^−/−^ cells display similar levels of alkylation-induced DNA breaks as wild type, PARP-1 activation is undetectable in AAG-deficient cells. Accordingly, *Aag*^−/−^ cells are protected from MMS-induced NAD^+^ depletion and glycolysis inhibition. MMS-induced mitochondrial dysfunction, however, is AAG-independent. Furthermore, treatment with FK866, a selective inhibitor of the NAD^+^ salvage pathway enzyme nicotinamide phosphoribosyltransferase (NAMPT), synergizes with MMS to induce cytotoxicity and *Aag*^−/−^ cells are resistant to this combination FK866 and MMS treatment. Thus, AAG plays an important role in the metabolic response to alkylation that could be exploited in the treatment of conditions associated with NAD^+^ dysregulation.

## Introduction

**Figure 1:**
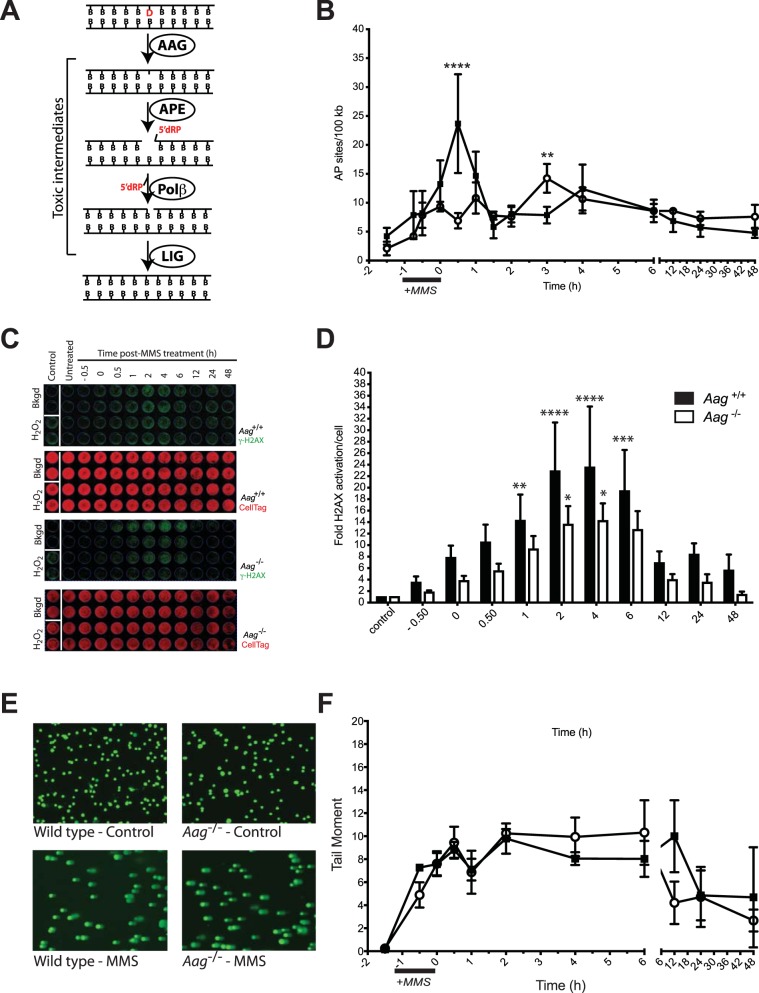
Alkylation damage is processed to toxic intermediates in *Aag*^−/−^ cells. (**A**) A diagram depicting the BER pathway initiated by AAG, showing key enzymatic steps and resulting toxic intermediates, i.e. abasic sites and strand breaks containing a 5’dRP moiety. (**B**) *Aag*^−/−^ cells display slower kinetics for AP site formation after treatment with MMS. Formation of AP sites over time following MMS exposure (2.5 mM) was measured in wild-type (filled squares) and *Aag*^−/−^ (open circles) MEFs. Significant differences between wild-type and *Aag*^−/−^ cells are indicated by **p ≤ 0.005, and ****p ≤ 0.0001. (**C)** Activation of histone H2AX over time following MMS exposure (2.5 mM) was measured in wild-type and *Aag*^−/−^ MEFs. H_2_O_2_ was used as a positive γH2AX inducer. Representative images of In-Cell Western plates for fluorescence staining intensity are shown, γH2AX (top, green), and total cell DNA staining by CellTag 700 (bottom, red). (**D**) Quantification of fluorescence staining intensity for γH2AX normalized to total cellular DNA. Bars represent means ± SEM of normalized staining intensities for at least 4 independent experiments. *p < 0.05, **p < 0.005, ***p < 0.001, and ****p < 0.0001. (**E**) Representative images obtained using the alkaline comet assay of wild-type and *Aag*^−/−^ MEFs untreated (control) or after exposure to 2.5 mM MMS. (F) DNA damage detected by alkaline comet analysis, expressed as tail moment, over time following exposure to 2.5 mM MMS in wild-type (filled squares) and *Aag*^−/−^ (open circles) MEFs. Values presented are the mean ± SEM of three independent experiments.

DNA base damage is unavoidable and abundant, arising from hydrolysis, oxidation, deamination and alkylation reactions^1^. Base damage arises both as a by-product of cellular metabolism and by exposure to environmental agents and has been associated with several pathologies such as cancer, chronic inflammation and neurodegeneration^2,3^. Chiefly dealing with endogenous DNA base lesions is the base excision repair (BER) pathway. Initiation of the BER pathway occurs via the removal of a damaged base by one of many glycosylases displaying substrate specificities. Base excision leads to the formation of abasic sites and strand breaks that are subsequently processed by downstream enzymes so that DNA synthesis and ligation can take place^4^ (Fig. 1A). Since many of the enzymatic steps taking place once BER is initiated give rise to repair intermediates with cytotoxic potential, balance and coordination at each step of the pathway is important for homeostasis and health^5–7^.

Alkyladenine DNA glycosylase, encoded by the *AAG* gene (also called *MPG* or *ANPG*, *Aag* in mice), is a key component of BER. AAG repairs alkylated DNA bases, deaminated purines, and exocyclic etheno DNA adducts, also displaying a low affinity for 8-oxoguanine, lesions that can be induced in response to alkylation, oxidative and/or inflammatory stress^8^. In support of a protective role for AAG against DNA base damage resulting from alkylation, oxidation and/or inflammation, *Aag*^−/−^ mice were found to be more susceptible than wild-type to chronic inflammation-induced DNA damage and display increased tumor multiplicity and severity after treatment with the alkylating agent azoxymethane in combination with the inflammation-inducing compound dextran sulphate sodium^9^. Contrasting with this protective role, additional studies showed that AAG drives alkylation-induced cytotoxicity in some cell types. *Aag*^−/−^ bone marrow myeloid cells are alkylation resistant if compared to wild-type bone marrow^10^, and *Aag*^−/−^ animals are protected from alkylation-induced toxicity in several tissues, most notably retina, cerebellum, thymus, pancreas and spleen^11,12^. In addition, AAG overexpression exacerbates alkylation-induced toxicity in numerous mammalian cell types and in mice^11–13^. More recently, AAG was also shown to drive cell death and tissue injury after ischemia-reperfusion^14^, a condition where a burst of reactive oxygen and nitrogen species occurs during the reperfusion phase. The diverse *in vivo* phenotypes associated with AAG deficiency in response to different stressors underline the importance of BER initiation and coordination for survival and raise questions on the cellular determinants affecting AAG-driven toxicity.

Successful BER additionally depends on the activation of poly(ADP-ribose) polymerase (PARP)-1, an NAD^+^ consuming enzyme which covalently modifies itself and other acceptor proteins with poly(ADP-ribose) (PAR), thereby promoting DNA repair protein recruitment to DNA damage sites^15^. In response to alkylation, excessive activation of PARP-1 is linked to excessive NAD^+^ consumption; a drop in cellular ATP levels and bioenergetic collapse^16^, and genetic deletion of *Parp-1* is protective against toxic insults in a variety of organ systems. *In vivo*, PARP-1 deficiency protects against alkylation-induced AAG-dependent cell death in several quiescent and rapidly dividing tissues^12^, but, long term, it does not suppress whole animal toxicity when BER is further imbalanced by increased AAG levels^17^. Thus, unrepaired SSB intermediates of AAG activity trigger cell death via PARP-1 activation, but preventing PARP-1 activation is not sufficient to ensure survival to alkylation when DNA damage levels are high.

Since PAR formation may depend on BER initiation and is sensitive to the availability of NAD^+^, we investigated a role for glycosylase-mediated BER in the metabolic response to DNA damage generated by alkylating agents. By carrying out a systematic evaluation of the downstream events to alkylation treatment in MEFs proficient or deficient in AAG activity, we characterize the consequences of alkylation damage in these cells. We show that AAG deficiency does not completely prevent the formation of alkylation-induced AP sites and DNA strand breaks but leads to a delay and a decrease in their production. However, PARP activation in response to alkylation is only detected in AAG-proficient cells. Consistent with a defect in PARP activation, *Aag*^−/−^ cells are protected from alkylation-induced NAD^+^ exhaustion and glycolysis inhibition, but still suffer mitochondrial dysfunction. Finally, we also show that further reduction of NAD^+^ levels by pharmacological inhibition of the NAD^+^ salvage pathway potentiates alkylation-induced cytotoxicity in AAG-proficient cells, while *Aag*^−/−^ cells are less vulnerable to this combination treatment. Taken together, our results suggest AAG plays an important role in regulating NAD^+^ levels and metabolic stress responses to alkylation. The importance of NAD^+^ metabolism in aging and its associated disorders, as well as in the response to chemotherapy, makes manipulating AAG activity, and therefore NAD^+^ concentrations, a potential attractive target for therapeutic efforts aimed at disease prevention and treatment.

## Results

### Alkylation DNA damage is processed in Aag-deficient cells

To analyze the downstream effects of AAG activity on alkylation-induced DNA damage, we initially monitored the levels of BER intermediate formation in AAG -proficient versus AAG -deficient cells after treatment with the model alkylating agent MMS, known to induce the AAG substrates 3-methyladenine (3meA) and 7-methylguanine (7meG). Following MMS treatment, repair intermediates were measured over a 48-hour period (Fig. 1).

MMS treatment induces AP sites in both wild-type and *Aag*^−/−^ cells, albeit with different kinetics (Fig. 1B). In wild-type cells, the peak of MMS-induced AP site formation is 30 minutes past MMS wash-out (p < 0.0001), while no significant AP site induction is seen at this same time point in *Aag*^−/−^ cells. The maximum AP site induction in the *Aag*^−/−^ MEFs was observed 3 hours post-MMS treatment (p < 0.004), a time where AP site levels were back to baseline in wild-type cells. These results indicate that while AAG is necessary for efficient alkylation base damage processing into AP sites, it is not essential, as AP sites are still formed after alkylation treatment in AAG -deficient cells. This is not unexpected, given that some alkylated bases may be prone to deglycosylation reactions that generate AP sites independently of repair processing^18^.

Next, we measured the levels of γH2AX, a marker for DNA damage and strand breaks, by In-Cell Western (see Supplementary Information for method) in MMS-treated wild-type and *Aag*^−/−^ MEFs. We find that there is a significant, albeit delayed, induction in the levels of γH2AX, measurable in *Aag*^−/−^ cells (Fig. 1C,D), consistent with the delay in AP site generation observed for *Aag*^−/−^ cells. We also employed the alkaline comet assay which detects a broad spectrum of DNA damage types, including AP sites, alkali-labile sites and both single and double strand breaks^19^. With this assay, we find overall damage load after alkylation treatment is similar in magnitude and kinetics in both wild-type and *Aag*^−/−^ cells (Fig. 1E,F), despite the differences in kinetics and magnitude in MMS induced AP-site generation. Tail moment increases rapidly 30 minutes into MMS exposure in both wild-type and *Aag*^−/−^ cells and remains increased up to 6 hours post-treatment. This observation suggests that some of the AAG-independent damage processing generates alkali-labile sites other than AP sites. Consistent with this idea, alkylation of the endocyclic nitrogen N7G generating 7meG, the dominant MMS-induced lesion, facilitates not only depurination but also ring-opening reactions, both of which can destabilize the base and induce strand cleavage under alkaline conditions^18,20^. Importantly, our results suggest that some of the MMS-induced repair intermediates are AAG-independent and while the overall quantity of MMS-induced damage is equivalent, the spectrum of repair intermediates may differ between genotypes. In order to shed more light into this issue, we also measured the levels of the DNA damage-responsive cyclin-dependent kinase inhibitor p21CIP1/WAF1 in wild-type and *Aag*^−/−^ cells, following MMS treatment. We find that p21CIP1/WAF1 is significantly induced after MMS treatment, and the response in *Aag*^−/−^ cells is indistinguishable from that of wild-type cells (Supplementary Fig. S1). Collectively, these results indicate that Aag deficiency alters but does not abolish alkylation damage processing into toxic AP sites and strand breaks.

### Aag knockout cells are defective in Parp activation after MMS treatment

Alkylation treatment is a potent activator of PARP-1^16,21^. Alkylation-induced PARP-1 activation is attributed to AAG-mediated generation of repair intermediates, such as AP sites and strand breaks, and AAG overexpression is associated with higher PARP-1 activation levels after alkylation treatment^22,23^. Our results showing repair intermediates are generated in AAG-deficient cells led us to re-examine the temporal relationship between repair intermediate formation and PARP-1 induction by examining the levels of PAR in MMS-treated wild-type and *Aag*^−/−^ MEFs.

**Figure 2:**
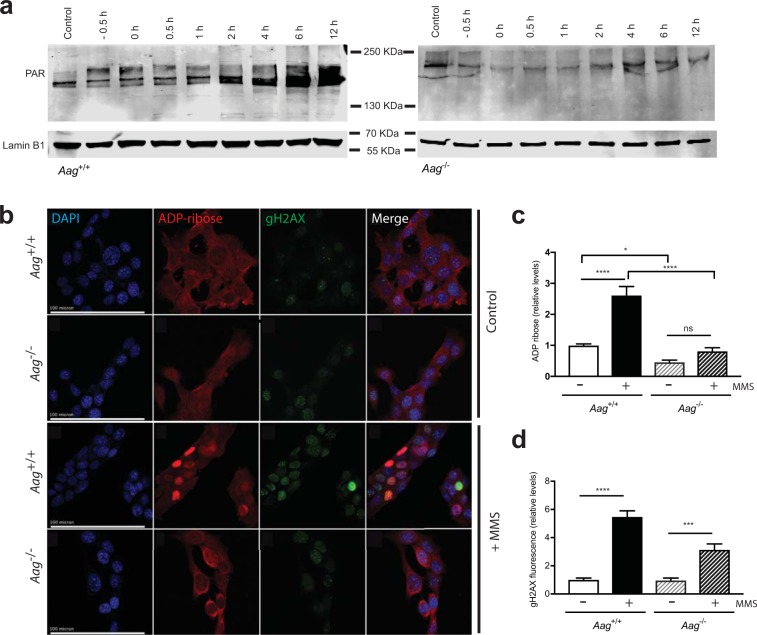
AAG is required for PARP activation following alkylation-induced strand break formation. **(a**) Representative immunoblots showing that MMS treatment (2.5 mM) induces PAR formation in wild-type MEFs and that the induction is absent in *Aag*^−/−^ MEFs. The nuclear lamin band shows protein loading. (**b**) ADP-ribose and γH2AX immunostaining in MEFs 2 hours after MMS treatment (1 mM). Scale bars, 100 μm. (**c**) Quantification of ADP ribose levels in wild-type and *Aag*^−/−^ MEFs treated or not with MMS (1 mM). (**d**) Quantification of γH2AX levels in wild-type and *Aag*^−/−^ MEFs treated or not with MMS (1 mM). Significant difference between cell types and treatments are indicated by *p ≤ 0.05; ***p ≤ 0.001, ****p ≤ 0.0001. In (**c**,**d**) bars and error bars indicate mean ± SEM for three independent experiments.

Alkylation treatment induces PAR formation in wild-type MEFs as determined by Western blotting (Fig. 2a) and immunofluorescence staining (Fig. 2b,c). Induction is seen 30 minutes into the one hour MMS treatment (−0.5 h) and steadily increases up to 12 hours after MMS treatment was removed, in agreement with the detection of strand breaks in wild-type cells (Fig. 2a). In our hands, MMS treatment leads to a >2.5-fold increase in PAR levels (p < 0.0001) in wild-type cells (Fig. 2c). On the other hand, no significant PARP activation was detected in MMS treated *Aag*^−/−^ cells (Fig. 2a,b). Quantification of PAR levels by immunofluorescence staining indicates that *Aag*^−/−^ cells are defective in inducing PAR formation after MMS treatment, and display a significant reduction in endogenous PAR levels (Fig. 2c). This defect in PAR formation is accompanied by a significant, albeit reduced, induction in the levels of γH2AX in MMS-treated *Aag*^−/−^ cells (Fig. 2d), in agreement with the In-Cell Western results (Fig. 1D). Thus, the PAR formation defect is notwithstanding the fact that *Aag*^−/−^ cells do generate toxic AP sites and strand breaks following MMS exposure, albeit at reduced levels and with altered kinetics.

### Alkylation damage processing and cellular bioenergetics

**Figure 3:**
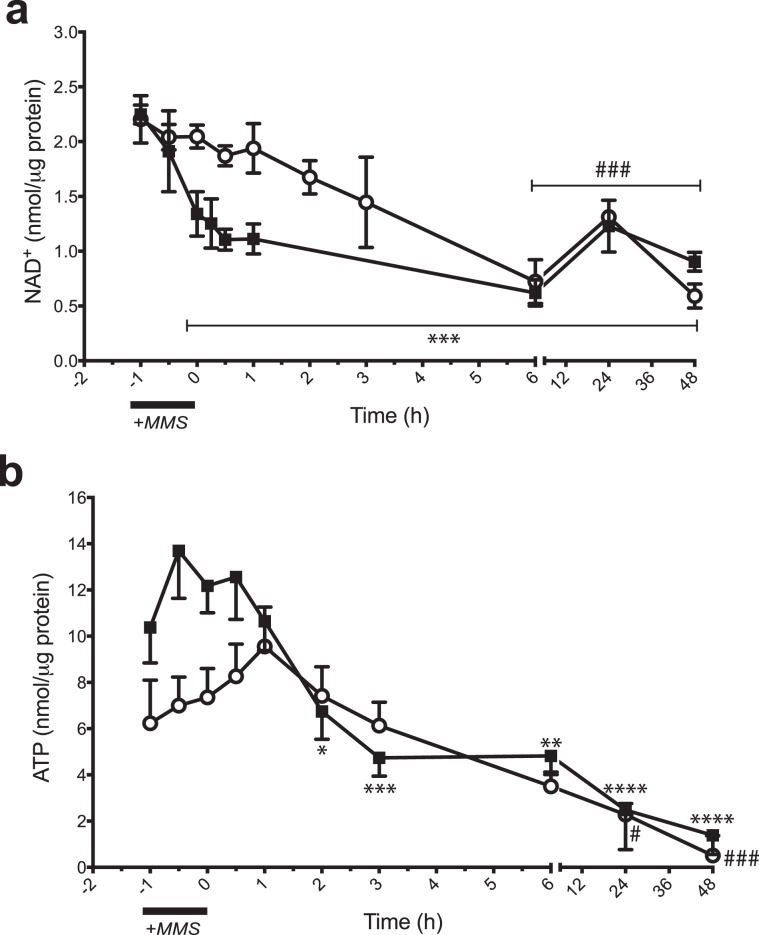
*Aag* knockout rescues NAD^+^ and ATP depletion at early time points after MMS treatment. (**a**) Cytosolic NAD^+^ levels in wild-type (black squares) and *Aag*^−/−^ (open circles) MEFs over time following MMS treatment. Values presented are the mean ± SEM of three independent experiments. (**b**) Cytosolic ATP measurements in wild-type (black squares) and *Aag*^−/−^ (open circles) MEFs over time following MMS treatment. Values presented are the mean ± SEM of six independent experiments. Significant changes versus baseline are indicated by *p ≤ 0.05, **p ≤ 0.01, ***p ≤ 0.001, and ****p ≤ 0.0001 for wild-type and #p ≤ 0.05, and ###p ≤ 0.001 for *Aag*^−/−^.

In order to monitor if the defect in PARP-1 activation seen in *Aag*^−/−^ cells alters the cellular metabolic landscape upon DNA damage induction, we measured NAD^+^ and ATP levels after MMS treatment in wild-type and *Aag*^−/−^ cells. We find that MMS treatment induced rapid NAD^+^ depletion in wild-type cells but that in *Aag*^−/−^ cells, NAD^+^ depletion was less marked and delayed (Fig. 3a). In the absence of MMS treatment, there was no observable difference in NAD^+^ levels between the two genotypes. In wild-type cells, MMS treatment leads to a significant reduction in NAD^+^ levels after 1 hour of MMS treatment (prior to MMS removal, our 0 h time point), when PAR formation is also evident by western blotting analysis, and with no recovery to pre-treatment levels up to 48 hours after MMS removal (all time points versus control, p < 0.001). In striking contrast, NAD^+^ levels are not significantly decreased in *Aag*^−/−^ cells in the initial time points post-treatment, consistent with the reduction in PAR formation in this genotype. However, at later time points, from 6 to 48 hours post-treatment, both genotypes displayed similar and significantly reduced levels of NAD^+^.

Next, ATP levels were monitored after MMS treatment (Fig. 3b). Surprisingly, we observed that control ATP levels appear to be reduced in untreated *Aag*^−/−^ cells compared with the wild type, although this difference is not statistically significant. In wild-type cells, the MMS treatment-induced NAD^+^ depletion precedes ATP depletion; wild-type ATP levels were significantly reduced 2 h (p = 0.03) after MMS washout, remaining significantly reduced up to 48 hours (p < 0.001) after treatment (Fig. 3b). In contrast, ATP levels remained unchanged or even slightly increased up to 3 hours after MMS treatment in *Aag*^−/−^ cells and there was no significant ATP depletion until the 6-hour time point. However, ATP levels were also significantly reduced in *Aag*^−/−^ cells at later time points (Fig. 3b, p = 0.02 for the 24 h and p = 0.0003 for the 48 h time point).

### Alkylation-induced mitochondrial dysfunction does not require Parp-1 activation

The experiments above show that AAG-deficient cells are protected from NAD^+^ and ATP depletion in the early time points after alkylation treatment, consistent with the defect in PARP activation observed in these cells. The metabolic effects of PARP activation have been previously studied and PARP inhibition had been previously shown to partially protect cells from alkylation-induced glycolytic defects and mitochondrial dysfunction^24^. This suggests PARP activation is required for DNA damage-dependent changes in glycolysis and mitochondrial function. However, PARP inhibition can have wide-ranging biological consequences^25^, and thus asking how alkylation alters the cellular metabolic landscape in AAG-deficient cells, where DNA damage is present but PARP activation not detectable becomes important. To address these questions we assessed oxidative phosphorylation and glycolysis in AAG-proficient and deficient cells treated or not with MMS.

Firstly, and given the apparent endogenous reduction in cytosolic ATP levels we observed in *Aag*^−/−^ cells (Fig. 3b), we investigated the basal metabolic profile of AAG-proficient and deficient MEFs (Supplementary Figs. S2 and S3). We find no statistically significant difference in basal metabolic function between genotypes, with both isogenic cell types displaying similar oxygen consumption rate (OCR, a measure of mitochondrial respiration, Supplementary Fig. S2) and extracellular acidification rate (ECAR, a measure of glycolytic function, Supplementary Fig. S3). This result indicates basal mitochondrial functions are not compromised as a result of *Aag* gene deletion; which is an important observation given the reported localization of the AAG protein to the mitochondria^26^.

**Figure 4:**
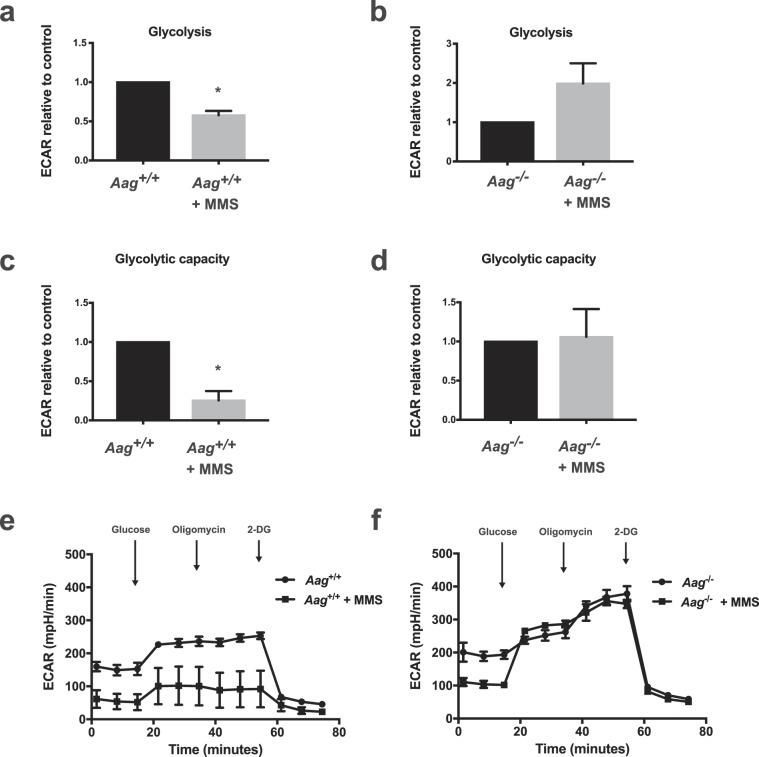
AAG deficiency rescues the glycolytic defects induced by MMS treatment in MEFs. (**a,b**) Glycolysis (ECAR after glucose) and (**c,d**) glycolytic capacity (ECAR after oligomycin) are significantly inhibited following MMS treatment (2.5 mM) in wild-type cells, shown in A and C, but preserved in *Aag*^−/−^ MEFs, shown in (**b,d**). Representative experiment of ECAR analysis in MMS-treated wild-type cells (**e**) and in MMS-treated *Aag*^−/−^ cells (**f**) (*p ≤ 0.05). Results represent the mean ± SEM of 3 independent experiments.

To determine whether AAG activity may modulate the effects of PARP-1 activation on glycolysis, we treated AAG-proficient and AAG-deficient MEFs with MMS for 1 hour and then measured glycolytic activity. Glycolytic function was evaluated by two parameters: (i) glycolysis, estimated by the ECAR reached by cells after addition of saturating amounts of glucose and (ii) glycolytic capacity, the maximum ECAR reached by cells following the addition of olygomicin, an inhibitor of oxidative phosphorylation driving cells to use glycolysis to its maximum capacity. Consistent with the reported PARP-dependent inhibition of glycolysis, MMS-treated AAG -proficient cells display a significant reduction in glycolysis and glycolytic capacity (Fig. 4a,c,e). Interestingly, in line with our finding that PARP activation does not occur after alkylation treatment in this genotype, AAG-deficient cells are completely protected from alkylation-induced glycolytic defects (Fig. 4b,d,f).

**Figure 5:**
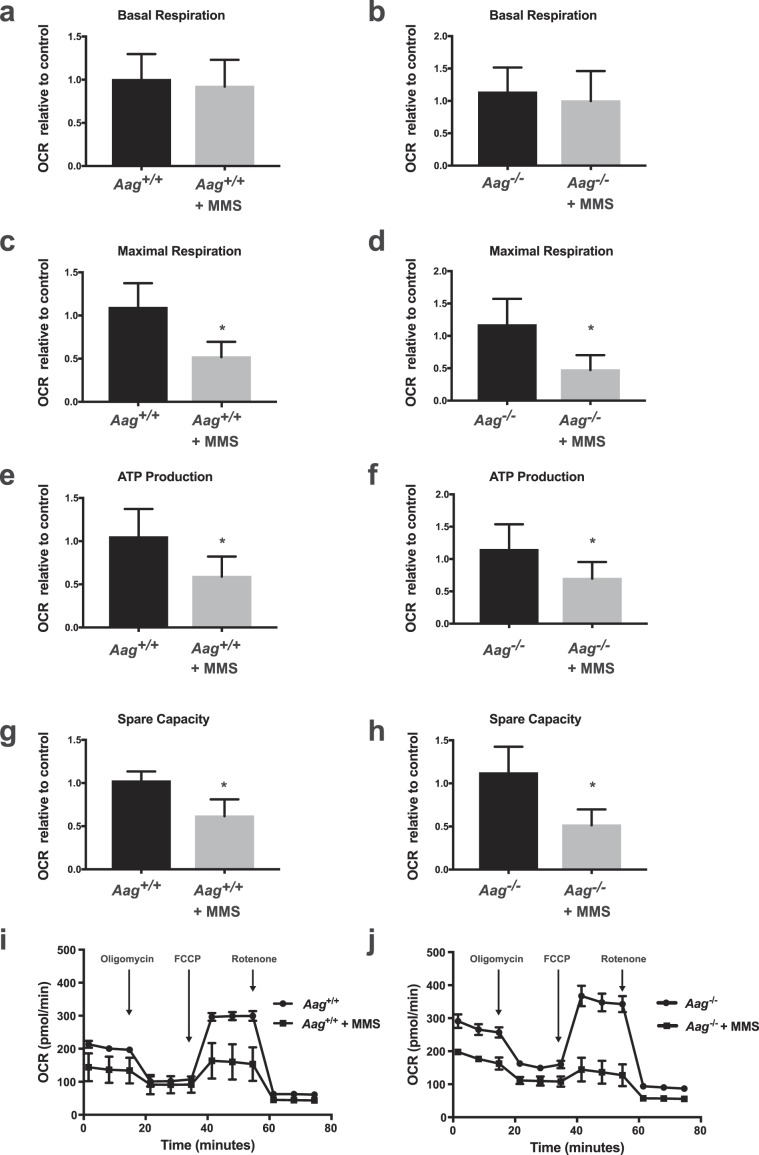
MMS induces mitochondrial dysfunction independent of the AAG genotype. Aspects of mitochondrial coupling and respiratory control measured by Seahorse extracellular flux analysis of OCR metabolic profile in MMS treated (2.5 mM) wild-type and *Aag*^−/−^ MEFs. Basal respiration (**a,b**), maximal respiration (**c,d**), ATP production (**e,f**) and spare respiratory capacity (**g,h**) in both wild-type and *Aag*^−/−^ cells. Representative cell respiratory control experiment in wild-type (**i**) and *Aag*^−/−^ (**j**) cells. *P ≤ 0.05. Results represent the mean ± SEM of 4 independent experiments.

Next, we assessed four parameters of cell respiratory control as a test of mitochondrial function, namely, basal respiration (oxygen consumption designed to meet cellular energy demands under baseline conditions), maximal respiration (the maximum OCR caused by the addition of a protonophoric uncoupler of the electron transport chain), ATP production (estimated by the decrease in respiration after inhibition of the ATP synthase by oligomycin) and mitochondrial spare capacity (a measure of how well the cell can respond to an increase in energy demand and calculated as the difference between the maximal respiration and the basal respiration). While MMS treatment did not significantly alter basal respiration; maximal respiration, ATP production and spare capacity were all significantly reduced after MMS treatment independently of AAG status (Fig. 5). That MMS induces mitochondrial dysfunction independently of AAG-initiated BER is supported by the fact that *Aag* wild type and *Aag*^−/−^ MEFs are equally sensitive to MMS treatment as evidenced by the MTS assay (Supplementary Fig. S4), an assay based on the reduction of the MTS tetrazolium compound and that can be used as an indicator of redox derangements and mitochondrial dysfunction^27^. These results thus suggest that mitochondrial function is affected by alkylation treatment through a mechanism that is independent of AAG and PARP-1.

### PARP-dependent NAD+ depletion synergises with NAMPT inhibition to induce cell death

**Figure 6:**
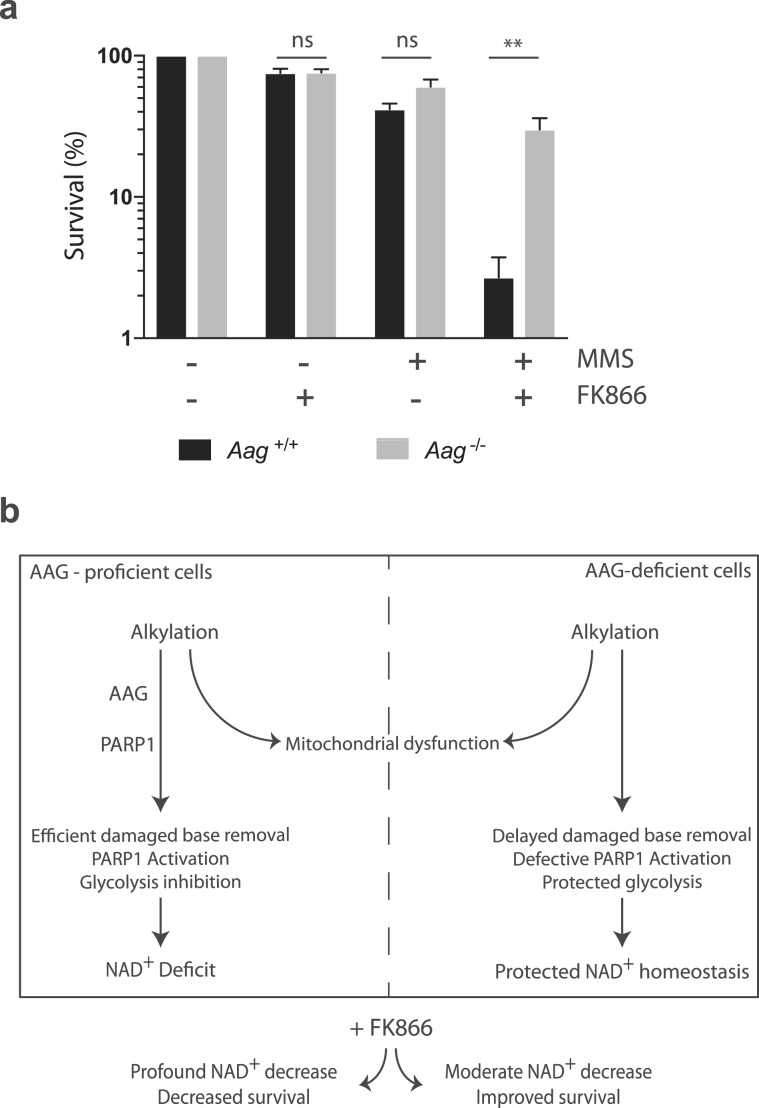
AAG-dependent alkylation-induced NAD^+^ depletion synergizes with NAMPT inhibition to increase cell death. (**a**) Clonogenic survival of wild-type (black bars) and *Aag*^−/−^ (grey bars) cells exposed to MMS (1 mM) in the presence or not of the NAMPT inhibitor FK866 (10 nM). Results represent mean ± SEM of 4 independent experiments; **p ≤ 0.01. (**b**) Model for the role of AAG-mediated BER in NAD^+^ homeostasis. In AAG-proficient cells, BER leads to efficient processing of alkylated bases and PARP activation, which results in disruptions to NAD^+^ homeostasis and cellular metabolism, including glycolysis inhibition and mitochondrial dysfunction. In AAG-deficient cells, alkylation-induced processing of alkylated bases is slower, resulting in the maintenance of NAD^+^ homeostasis and glycolysis but no rescue from mitochondrial dysfunction.

NAMPT regulates the intracellular NAD^+^ pool by catalysing a rate limiting step in the mammalian NAD^+^ salvage pathway^28^. Since we have shown that AAG-deficient cells are protected against alkylation-induced NAD^+^ depletion, we next investigated whether AAG status would modulate susceptibility to NAMPT inhibition. We treated cells with MMS in the presence or absence of FK866, a selective small molecule inhibitor of NAMPT. The dose of FK866 we used (10 nM) was effective in significantly depleting NAD^+^ pools in both wild-type and *Aag*^−/−^ MEFs (Fig. S5) but did not lead to a decrease in viability (Fig. 6a). However, the combination of sub lethal doses of FK866 and MMS leads to a dramatic reduction in survival in wild-type MEFs. Strikingly, *Aag*^−/−^ cells are significantly more resistant to the MMS and FK866 combination treatment (p < 0.01). We therefore propose that AAG-mediated BER plays a critical role in cell fate upon alkylation, connecting DNA repair and metabolism (Fig. 6b). Taken together, these results suggest Aag-activity on alkylated substrates contributes to the cellular ability to survive perturbations in the NAD^+^ pool that could influence cellular outcome in a number of pathophysiological scenarios.

## Discussion

Previous work has identified the initiation of the BER pathway by the AAG enzyme as playing a cytotoxic role during tissue repair in response to alkylation damage (reviewed in^8^) and ischemia/reperfusion^14^. In an effort to understand how AAG-initiated BER modulates different stages in the cellular response to base damage, we examined BER intermediate generation, PARP-1 activation levels and aspects of cellular metabolism, after alkylation treatment in AAG-proficient or deficient MEFs. We find that MMS-induced AP-site and strand break formation occurs in both AAG-proficient and AAG-deficient MEFs but that *Aag*^−/−^ cells are defective in PARP-1 activation in response to MMS treatment. Importantly, the diminished PARP activation observed in MMS-treated *Aag*^−/−^ cells is accompanied by protection against NAD^+^ depletion and against glycolytic defects but not against mitochondrial dysfunction. Finally, we find that further perturbations in the NAD^+^ pool by treatment with the NAMPT inhibitor FK866 sensitize wild-type cells to MMS while *Aag*^−/−^ cells are significantly protected from the FK866 and MMS combination treatment. Together, our results are consistent with a model where, in the absence of AAG, alkylated bases are still processed into toxic strand break intermediates but with delayed kinetics (Fig. 6). The delayed damaged base removal is associated with defective PARP-1 activation and protected NAD^+^ homeostasis and glycolysis but similarly altered mitochondrial function (Fig. 6). Through its role driving PARP activation and modulating NAD^+^ levels, AAG may play an important role in the metabolic vulnerabilities in response to alkylation that could be exploited therapeutically, especially within regimens targeting the manipulation of NAD^+^ concentrations.

The BER pathway is largely responsible for removing numerous aberrant bases generated by oxidation, deamination and alkylation reactions, which can occur spontaneously or be environmentally induced. Because such base damage can be cytotoxic and/or mutagenic, BER acts to minimise detrimental outcomes associated with base damage. Indeed, defects in glycosylases such as OGG1 and UNG that recognise oxidised bases or uracil, respectively, lead to increased susceptibility to tissue damage after exposure to conditions inducing oxidation and deamination of bases, including ischemia-reperfusion^29,30^. In contrast, defects in AAG are instead associated with protection against tissue damage induced by alkylation or ischemia-reperfusion^12,14^. How AAG-initiated BER promotes tissue damage has not been fully elucidated, but it has been proposed that AAG-dependent generation of toxic BER intermediates such as AP sites and strand breaks underlies this toxicity. In agreement, AAG deficiency is associated with decreased AP site formation in response to alkylation^11^, and also in response to ischemia-reperfusion^14^. Our results also indicate that MMS treatment induces a more rapid and more pronounced accumulation of AP sites in AAG-proficient cells than in AAG-deficient cells, consistent with the ability of AAG to excise alkylated DNA bases. However, MMS-induced AP site and strand break generation is not abolished in *Aag*^−/−^ MEFs. Consistent with this observation, Smith and Engelward^31^ reported that the alkylated bases 3meA and 7meG, both AAG substrates generated from MMS treatment, are removed from the genome of AAG-deficient embryonic stem cells, with slower kinetics for 3meA but comparable kinetics for 7meG. In addition, Parrish *et al*. (2018) also reported an increase in repair intermediates following MMS challenge in *Aag*^−/−^ MEFs^32^. Taken together, these results indicate that MMS-induced repair intermediates can be AAG-independent, potentially generated by spontaneous depurination of alkylated DNA^18^ and/or the action of redundant repair glycosylases that could also process AAG substrates.

Consistent with our results for AP sites, we also observe a difference in post-MMS γH2AX kinetics between wild type and *Aag*^−/−^ cells. It is important to mention that while 7meG is the predominant MMS-induced adduct, it is considered to be non-toxic. 3meA, on the other hand, while a minor adduct, is a replication-blocking lesion^31,32^. In wild type cells, once BER is initiated, the increased and rapid formation of AP sites would trigger a rapid block in DNA synthesis, replication fork collapse and strand break generation^5^. In *Aag*^−/−^ cells, unrepaired 3meA lesions would slow down the progression of the replication fork in a checkpoint independent fashion and favour a lesion bypass mechanism^5^. Additionally, clustered AP sites within nucleosome core particles have been shown to accumulate DNA–protein cross-links, and produce significant amount of strand breaks^33^. Therefore, the difference in post-MMS γH2AX kinetics between wild type and *Aag*^−/−^ cells is consistent with the delay in AP site formation observed in *Aag*^−/−^ cells.

SSB induce toxicity in both replicating and non-replicating cells through the activation of PARP-1 via PAR synthesis^34^. PARP-1 catalytic activity and PAR synthesis is stimulated upon PARP-1 binding to damaged DNA, including sites of SSB but how PARP-1 coordinates cellular responses, including DNA repair, to different types of stress stimuli is still unclear. Here we show that AAG-deficient MEFs do not accumulate PAR in response to MMS treatment, despite evidence for MMS-induced strand break generation in this genotype. This supports the idea that PARP-1 would have specific requirements for recognition of strand breaks and that AAG-dependent BER initiation would be necessary to generate BER intermediates that can be efficiently recognised by PARP-1. Consistent with this, PARP-1 was shown to have specific affinity to AP sites and to nicked DNA containing a 5’deoxyribose phosphate (5’dRP)^23,35^, two intermediates generated as a result of the action of a monofunctional glycosylase such as AAG. 5’dRPs have shown to be especially toxic^5^. Thus, Aag-initiated BER could be notably toxic because it generates intermediates that are particularly effective in activating PARP-1.

Free or protein-bound PAR polymers act as signal transducers that mediate a plethora of cellular responses to a wide variety of stress stimuli^36^. Chiefly among these cellular responses are alterations in cellular metabolism; PAR production depends on NAD^+^ availability thus PARP-1 activation is linked to the maintenance of NAD^+^ levels and metabolic homeostasis. In response to alkylation stress, PARP-1 activation induces both glycolysis inhibition and mitochondrial dysfunction. Glycolysis inhibition was shown to be a consequence of direct inhibition of hexokinase by PAR, while deterioration of mitochondrial function was proposed to result from the reduced availability of glycolytic substrates due to PAR-mediated glycolysis inhibition^24,37^. Indeed, alkylation-induced mitochondrial dysfunction was partially rescued by PARP inhibition or by supplementation with pyruvate, a glycolysis-derived substrate for oxidative phosphorylation^24^. Our results confirm that PARP-1 activation is required for alkylation-induced glycolysis inhibition; a defect in PARP-1 activation in *Aag*^−/−^ cells is coupled to protection against MMS-induced NAD^+^ depletion and glycolysis inhibition. In stark contrast, *Aag*^−/−^ cells experience the same levels of MMS-induced mitochondrial dysfunction as wild-type cells, and are not protected from NAD^+^ depletion and ATP exhaustion at later time points after MMS treatment. This result suggests therefore that alkylation-induced mitochondrial dysfunction can occur independently from PARP-mediated NAD^+^ depletion and glycolysis inhibition.

NAD^+^ is a vital coenzyme for redox reactions and an important co-substrate for enzymes involved in the regulation of key cellular processes^38,39^. Therefore, Aag-dependent modulation of NAD^+^ levels in response to alkylation could have a major impact in the physiology of NAD^+^ metabolism and signalling, and may create a metabolic vulnerability that can be exploited therapeutically. The NAD^+^ salvage pathway enzyme NAMPT inhibitor FK866 is an attractive strategy to clinically modulate NAD^+^ levels^40^. Pharmacological inhibition of NAMPT by FK866 was previously shown to sensitize immune cells to the alkylating agent 1-methyl-3-nitro-1-nitrosoguanidine (MNNG)^41^ and glioblastoma cells to the alkylating agent temozolomide^42,43^. In agreement, we show that FK866 sensitises MEFs to MMS-induced cell death. However, that this depends on AAG-mediated repair emphasises the important role played by AAG-initiated BER in regulating cellular sensitivity to alkylation-induced NAD^+^ depletion and metabolism. Given the wide substrate-specificity of the AAG enzyme, further studies are required to examine the role of AAG in regulating cellular resistance to NAD^+^-depleting situations resulting from other sources of genotoxic stress, such as oxidative stress and chronic inflammation.

Overall, our results shed light on the metabolic consequences of alkylation treatment and indicate that AAG plays an important role in cellular resistance to alkylation-induced NAD^+^-depletion. AAG activity levels were shown to widely vary in the human population^12^. In addition, elevated AAG activity has been associated with detrimental health outcomes, such as increased cancer risk^44,45^ or poorer overall survival in cancer patients^46^. It is not clear whether the role played by AAG in maintaining NAD^+^ concentrations in response to genotoxic stress significantly contributes to this reported association. Nevertheless, given the increased interest in NAD^+^ biology and its therapeutic manipulation for disease prevention and treatment, a better understanding of the relationship between AAG-initiated BER and the regulation of NAD^+^ levels and cellular energy metabolism is warranted.

## Methods

### Mouse cell lines and culture

The *Aag*^−/−^ mice have been described^47^ and were a kind gift from Prof. Leona D. Samson (MIT, Cambridge, MA, USA). Mice were on a C57BL/6J background and used to generate primary MEFs for the study, as described in^48^. MEFs were cultured in DMEM supplemented with 10% FBS, L-glutamine and penicillin/streptomycin. MEFs were incubated at 37 °C in a humidified atmosphere under 5% CO_2_ and normoxic conditions. Because MEFs undergo premature senescence when grown under normoxic conditions, all experiments were performed using low-passage cells (passage ≤ 5). Primary MEFs used were derived from at least three independent isolations, and experiments were conducted using littermate wild-type controls. In addition, transformed MEFs, a generous gift from Prof. Leona D. Samson, were used for clonogenic experiments. Animals were provided with food (transgenic mouse diet, B & K Universal Ltd, Hull, UK) and water *ad libitum*. All methods were carried out in accordance with relevant guidelines and regulations from the University of Surrey. All experimental protocols were approved by the University of Surrey Animal Welfare Ethical Review Body (AWERB) and carried out under UK Home Office License in accordance with the UK Animals (Scientific Procedures) Act 1986. Methyl methanesulfonate (MMS), FK866 hydrochloride hydrate, deferoxamine mesylate salt, and erythro-9-(2-hydroxy-3-nonyl)-adenine (EHNA) hydrochloride salt were obtained from Sigma.

### Experimental treatments

Wild-type and *Aag*^−/−^ MEFs were treated with MMS (in serum-free media, 1 hour), and incubated in drug-free complete media for the indicated repair periods at 37 °C. MMS dose was 2.5 mM, unless otherwise indicated. For the control group, cell harvesting was performed 48 hours after the replacement of serum-free media with complete media. The NAMPT inhibitor FK866 was diluted in DMSO and used at a final concentration of 10 nM.

### Apurinic/apyrimidinic (AP) site quantification

Genomic DNA isolation for AP site quantification was performed using the Roche DNA isolation kit (Roche) in the presence of EHNA hydrochloride (5 µg/ml) and deferoxamine (66 µg/ml). All steps were carried out at 4 °C to avoid heat-induced AP site formation. Quantification of AP sites was performed using the Dojindo DNA damage quantification kit (Dojindo), as per manufacturer’s instructions. AP sites were normalized to the concentration of aldehyde reactive probe (ARP)-labeled DNA as quantitated by fluorimetric measurement of SYBR Green-labeled DNA.

### Comet assay

MEF cells (2.5 × 10^5^ cells/sample) were treated with MMS as described. Treated cells were mixed with warm 0.5% UltraPure Low Melting Point Agarose (LMPA) (Invitrogen) and layered onto pre-chilled comet glass slides (Trevigen). Slides were immersed in pre-chilled comet assay lysis solution (Trevigen) overnight at 4 °C. After lysis, comet slides were immersed into freshly prepared alkaline solution (0.3 M NaOH, 100 mM EDTA, pH 13) for at least 1 hour at room temperature. Electrophoresis was in Tris/Borate/EDTA (TBE) buffer at 19 V (1 V/cm) for 10 min. After electrophoresis, slides were fixed in 70% ethanol for 5 minutes, air-dried and DNA was stained with SYBR Green I for 30 min. DNA strand breakage was expressed as “comet tail moment”, which is the product of the tail length and the fraction of DNA that has exited the nucleus during electrophoresis. The comet tail moment was measured for at least 100 cells per sample using the ImageJ plugin OpenComet (version 1.3) from images taken on EVOS FL Cell Imaging System (Life Technologies).

### Western blotting

Treated cells were lysed in RIPA buffer (Sigma). Lysates (20 μg) were resolved by SDS-PAGE and transferred to PVDF membranes using the Trans-Blot Turbo Transfer System (Bio-Rad). The blots were probed with polyclonal rabbit anti-PAR antibody (1:500, BD Pharmingen) and anti-lamin B1 antibody (1:2000, Abcam), followed by detection with IRDye 800CW goat anti-rabbit IgG and IRDye 680RD goat anti-mouse IgG (LI-COR Biosciences). Membranes were scanned using an Odyssey CLx infrared imaging system (LI-COR Biosciences).

### Immunofluorescence

Cells were cultured directly onto Nunc Lab-Tek II 8 Chamber Slides (Thermo Scientific) and treated in serum-free medium for 1 hour, in the presence or not of MMS (1 mM). After treatment, cells were incubated in fresh complete medium for 1 hour and subsequently fixed in 4% paraformaldehyde. Fixed cells were permeabilised using a 1:1 methanol:acetone solution for 10 minutes at room temperature. Cells were then blocked for 1 hour with 10% normal goat serum (NGS) and labeled using anti-γH2AX phospho S139 (1:500, Abcam) antibody and an anti-pan-ADP-ribose binding reagent (1:1000, Millipore), at 4 °C overnight. Primary antibodies were prepared in 1% NGS to reduce background staining. Slides were washed with TBS, 0.1% Tween-20 prior to incubation with secondary antibodies. Secondary antibodies used were goat anti-rabbit DyLight 488 (Vector Laboratories) and goat anti-mouse DyLight 594 (Vector Laboratories), 1:1000 in TBS containing 1% NGS. All incubation steps were performed protected from light in a humidified chamber. Slides were mounted using Fluoroshield (Sigma). Samples were imaged using a Nikon A1M laser scanning confocal microscope and DS-Qi1 Widefield Camera (on an Eclipse Ti-E microscope). Fluorescence intensity measurements were performed using Fiji (NIH, Bethesda, MD).

### Measurements of NAD^+^ and ATP levels

Cytosolic NAD^+^ levels were measured using the NAD^+^/NADH cell-based assay kit (Cayman Chemical Company), following manufacturer’s instructions. Cytosolic ATP levels were measured using the ATP determination kit (Life Technologies), following manufacturer’s instructions.

### Mitochondrial function

Mitochondrial function was determined using a Seahorse XFp Analyzer (Agilent). Briefly, wild-type and *Aag*^−/−^ cells were seeded at 1 × 10^4^ cells per well into Seahorse Bioscience XFp cell culture plates and cultured overnight as described above. The following day, cells were treated with MMS for 1 hour. After this period, MMS-containing media was replaced with Seahorse XF base medium (pH 7.4) containing 10 mM glucose, 2 mM glutamine and 1 mM sodium pyruvate and cells incubated for 45 minutes at 37 °C without CO_2_. Oxygen consumption rate (OCR) was determined under basal and MMS-treatment conditions followed by the sequential addition of oligomycin (1 mM), carbonyl cyanide-4-(trifluoromethoxy) phenylhydrazone (FCCP, 1 mM), as well as rotenone/antimycin A (0.5 mM). This allowed for an estimation of the contribution of individual parameters for basal respiration, maximal respiration, spare respiratory capacity, and ATP production. Normalisation to cell number in the well following the experiment was used to control for variation in plating between different genotypes.

### Measurements of glycolysis as ECAR

Glycolysis as extracellular acidification rate (ECAR) was measured using a Seahorse XFp Analyzer (Agilent) as described above for the mitochondrial function assays, with the following differences: (i) after MMS treatment media was replaced with Seahorse XF base medium (pH 7.4) solely supplemented with 2 mM glutamine; (ii) ECAR was measured under basal and MMS treated conditions followed by the sequential addition of 10 mM glucose, 0.5 mM oligomycin, and 100 mM 2-Deoxy-D-glucose, for estimation of the contribution of individual parameters for glycolysis and glycolytic capacity.

### Clonogenic assay

Clonogenic survival assays were performed as described^49^. Briefly, transformed MEFs were seeded in triplicate into 6-well plates, and incubated with complete DMEM containing or not FK866 (10 nM). Twenty-four hours later, cells were subsequently treated with MMS (1 mM) for 1 hour at 37 °C in serum-free media containing the appropriate concentration of FK866. Following MMS treatment, cells were incubated in complete DMEM for 5 to 7 days. Colonies were fixed with methanol and stained with 0.1% crystal violet. Colonies containing >50 cells were scored and the percent survival calculated relative to untreated control.

### Statistical analysis

Datasets were compared by either one-way ANOVA (genotype effect) or two-way ANOVA (genotype and treatment effect) with Sidak’s or Dunnett’s multiple comparison test, as appropriate. For the metabolic flux experiments, significance level was determined by performing ANOVA on the complete data set with Tukey’s post-hoc testing. The level of statistical significance was set *a priori* at p < 0.05. GraphPad Prism version 7 was used for statistical analysis.

## Supplementary information

Supplementary information.
