# Supplementary material for: Alkyladenine DNA glycosylase deficiency uncouples alkylation-induced strand break generation from PARP-1 activation and glycolysis inhibition
Source: Sci Rep. 2020 Feb 10;10:2209. doi: 10.1038/s41598-020-59072-6 (PMC7010680; doi:10.1038/s41598-020-59072-6)
Supplement: Supplementary file 1 — Supplementary information. [file 41598_2020_59072_MOESM1_ESM.pdf]

Supplementary information

Manuscript title: Alkyladenine DNA glycosylase deficiency uncouples alkylation-induced strand break generation from PARP-1 activation and glycolysis inhibition.

Author list: Fahad A. Alhumaydhi, Debora de O. Lopes, Diana L. Bordin, Abdullah S. M. Aljohani, Cameron B. Lloyd, Michael D. McNicholas, Larissa Milano, Clara F. Charlier, Izabel Villela, João Antonio P. Henriques, Kathryn E. Plant, Ruan M. Elliott and Lisiane B. Meira

Alhumaydhi et al.
Supplementary Methods

In Cell Western for γ-H2AX quantification

Cells were seeded at 10,000 cells/well in a clear-bottomed black 96-well plate (Nunc International, Rochester, NY) and treated with MMS (2.5 mM) or H_2O_2 (1 mM). Each time point was carried out in quadruplicate, and four independent experiments were performed. Upon completion of treatment, culture medium is aspirated and cells immediately fixed with 4% paraformaldehyde for 20 minutes at RT. Cells are then washed with PBS and permeabilized with 0.1% Triton X-100 in PBS for 3 x 5 minutes at RT with gentle shaking. Next, cells are blocked with Odyssey Blocking Buffer for 1.5 hours at RT with gentle shaking, followed by overnight incubation with 1:200 phospho-histone H2AX (Ser139) (20E3) rabbit monoclonal antibody (Cell Signaling Technology, London, UK), at 4 °C with no shaking. Primary antibody incubation was followed by detection with IRDye 800CW Goat anti-Rabbit secondary antibody (1:1000, Li-Cor, Lincoln, NE). CellTag 700 Stain (1:10,000, Li-Cor, Lincoln, NE) was used for normalization to cell number. Plates were imaged using an Odyssey instrument (Li-Cor, Lincoln, NE).

Cdkn1a (p21CIP1/WAF1) RT-qPCR.

The cells were harvested for RNA isolation using the PureLink RNA Mini kit (Thermo Fisher Scientific, UK) according to the manufacturer's instructions. First strand cDNA synthesis was carried out using the Maxima First Strand cDNA Synthesis with dsDNAse (Thermo Fisher Scientific, UK) using with oligo (dT) primers and following the manufacturer's instructions. All RT-qPCR reactions

were singleplexed in a 96-well plate (triplicate wells), using SYBR Green Luminaris Color HiGreen qPCR Master Mix, low ROX (Thermo Fisher Scientific, UK). The amplification was done using the following cycling conditions: 50ºC/2 minutes, 95ºC/10 minutes, followed by 40 cycles of 95ºC/15 seconds and 60ºC/1 minute. At the end of the reaction, an additional cycle for melting curve analysis was added to confirm primer specificity. Expression levels were quantified as fold-expression using mouse β-actin (Actb NM_007393) as reference gene (ΔΔCt). The primers used were: Actb_F: 5'-CATTGCTGACAGGATGCAGAAGG-3', Actb_R: 5'-AGGTCTTTGCGGATGTCCACGT-3' for *Actb* (NM_007393) and Cdkn1a_F: 5' – TCGCTGTCTTGCACTCTGGTGT-3', Cdkn1a_R: 5'-CCAATCTGCGCTTGGAGTGATAG-3' for the *Cdkn1a* (p21CIP1/WAF1) gene (NM_001111099).

MTS assays

Primary Aag wild type and Aag knock-out MEFs were seeded at a density of 1 x 10^4 cells/well. Cell survival was determined 48 h after treatment using the MTS-based CellTiter 96® AQueous One Solution Cell Proliferation Assay (Promega).

Alhumaydhi et al.
Supplemental Figures

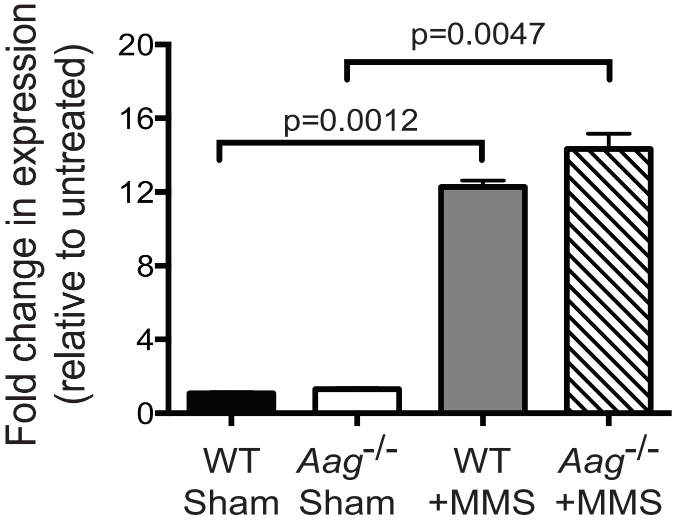

Supplemental Figure 1 - MMS treatment induces the expression of p21CIP1/WAF1 in both wild type and *Aag* knockout MEFs. Quantitative PCR (qPCR) was used to quantify p21CIP1/WAF1 induction after MMS treatment. The values are fold change in expression relative to untreated, n=3 experiments, error bars represent SEM, statistical significance assessed by unpaired t-test analysis.

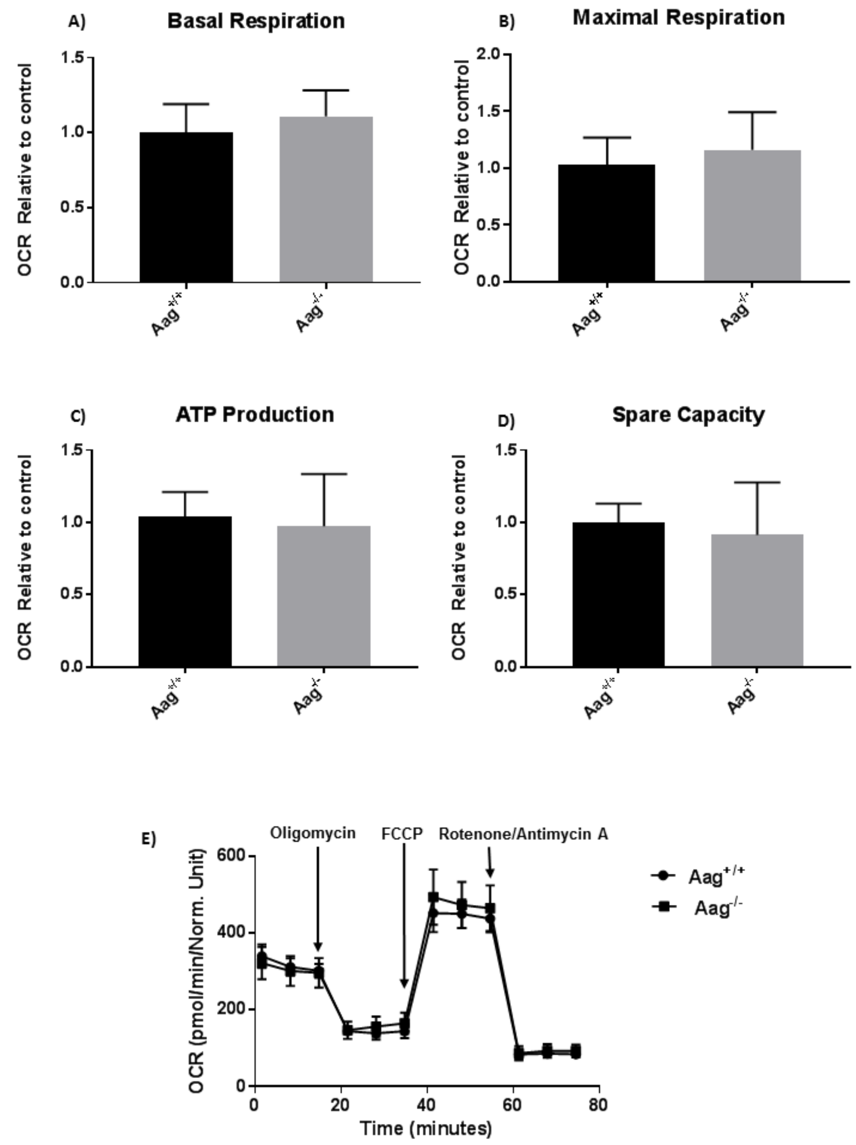

Supplemental Figure 2 – Aag status does not alter basal mitochondrial function. Analysis of mitochondrial OCR in untreated *Aag* $^{+/+}$ and *Aag* $^{-/-}$ MEFs . Basal OCR (A), maximal OCR (B), ATP production (C) and spare capacity (D) of *Aag* $^{-/-}$ cells as relative to wild type. (E) A representative respiratory control profile is shown. Experiments were repeated four times.

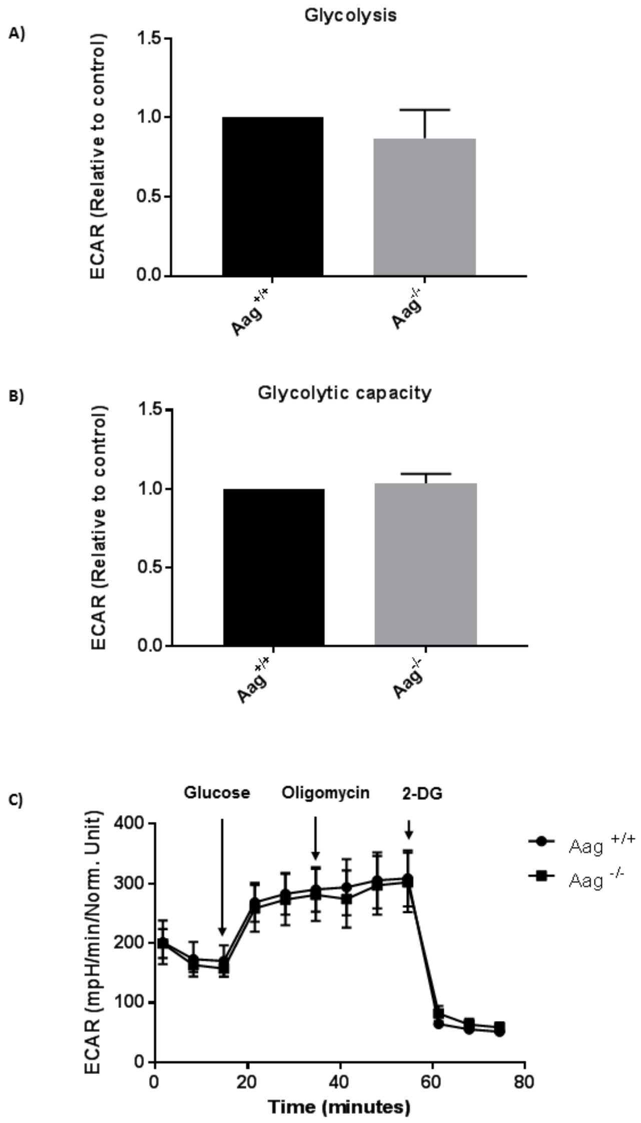

Supplemental Figure 3 – Aag status does not alter basal glycolysis. The results were obtained by measuring the extracellular acidification rate (ECAR) of cells. Sequential compound injections measured: (A) glycolysis, (B) glycolytic capacity. Graphs were produced using results from 3 independent experiments. (C) Representative experiment showing the basal glycolytic profile of *Aag*$^{+/+}$ and *Aag* $^{-/-}$ cells.

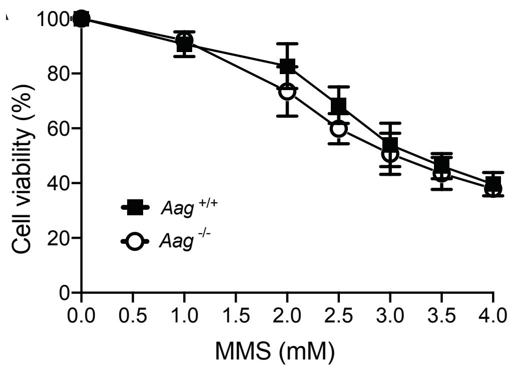

Supplemental Figure 4. Cell viability in response to alkylation treatment: *Aag*$^{+/+}$ (filled squares) and *Aag*$^{-/-}$ (open circles) MEFs were exposed to the alkylating agent MMS and viability assessed by the MTS growth inhibition assay, which can also be used as an indicator of redox derangements and mitochondrial dysfunction. Values presented are the mean ± SEM of at least three independent experiments.

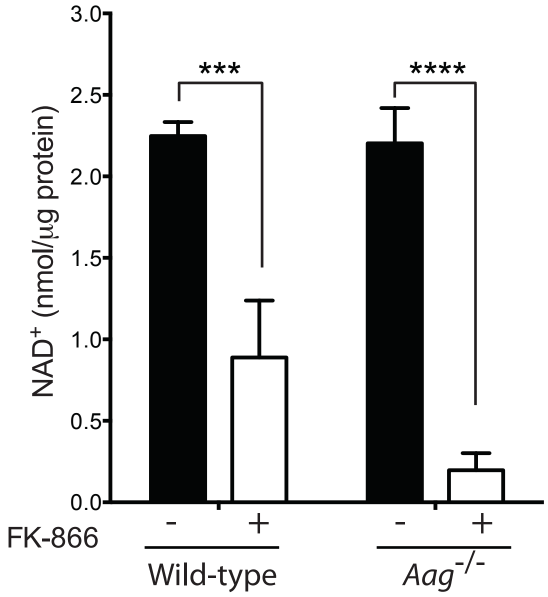

Supplemental Figure 5. Cytosolic NAD+ measurements in *Aag*+/+ and *Aag*-/- MEFs treated with FK866 (10 nM). Values presented are the mean ± SEM of three independent experiments; ***, p<0.001, and ****, p<0.0001.

Panel A - Wild-type

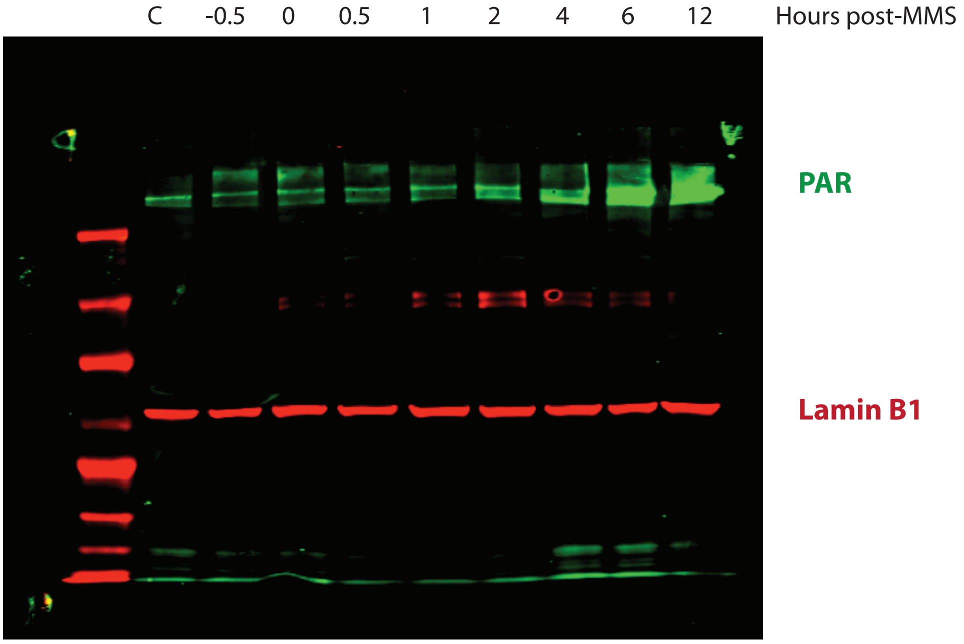

Panel B - *Aag* knock-out

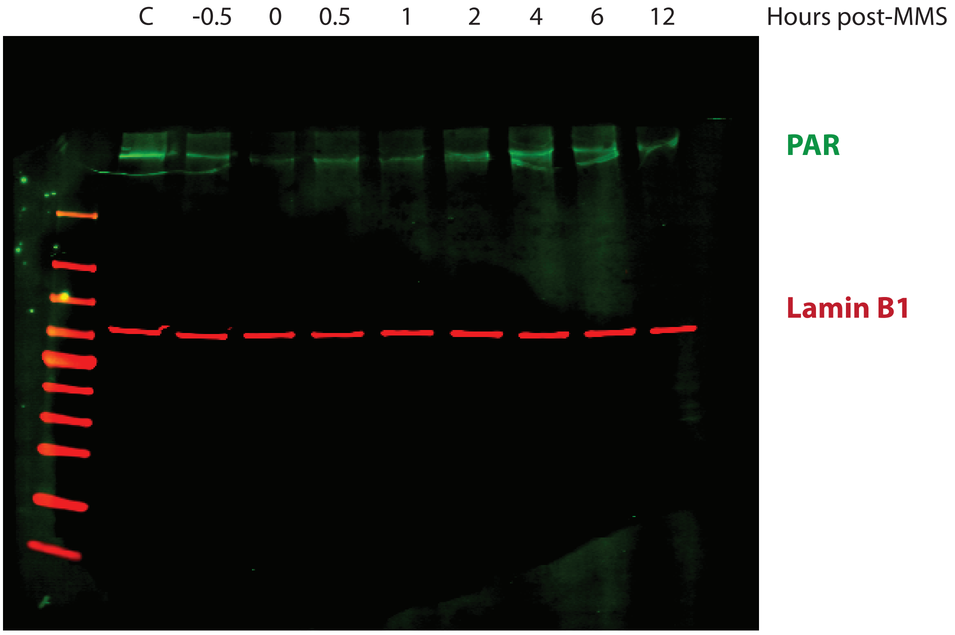

Supplemental Figure 6 - Original images of the representative immunoblots shown in Figure 2.
